# Cooking behavior among mothers of children aged 2–5 years old in Kendari, Southeast Sulawesi, Indonesia

**DOI:** 10.1186/s12889-024-17826-1

**Published:** 2024-02-06

**Authors:** Intan R. Nirmala, Judhiastuty Februhartanty, Rina Agustina, Rini Sekartini

**Affiliations:** 1 Department of Nutrition, Faculty of Medicine, Universitas Indonesia - Dr. Cipto Mangunkusumo General Hospital, Jakarta, Indonesia; 2 Department of Nutrition, Health Polytechnic of Kendari, Ministry of Health, Kendari, Indonesia; 3 Southeast Asian Ministers of Education Organization Regional Centre for Food and Nutrition (SEAMEO-RECFON)/PKGR Universitas Indonesia, Jakarta, Indonesia; 4 Human Nutrition Research Center, Indonesian Medical Education and Research Institute (HNRC-IMERI), Faculty of Medicine, Universitas Indonesia , Jakarta, Indonesia; 5 Department of Pediatric, Faculty of Medicine, Universitas Indonesia - Dr. Cipto Mangunkusumo General Hospital, Jakarta, Indonesia

**Keywords:** Cooking, Children 2–5 years old, Indonesia, Qualitative research

## Abstract

**Background:**

Cooking at home was associated with parental feeding practices. This study aimed to explore the interplay of components in cooking behavior of mothers with young children aged 2–5 years old in Kendari city of Southeast Sulawesi, Indonesia.

**Methods:**

This qualitative study involved 33 mothers from Kendari city, which was represented by each two sub-districts in coastal and mainland areas that were randomlyselected. Six focus group discussions (FGDs) were carried out using 20-item guide questions. The guide questions were developed following the Model of Goal-Directed Behavior (MGDB) theory approach exploring the components of cooking behavior i.e., skill, intention, desire, confidence, and attitude in cooking. Data analysis was performed in the field to assist decision on data saturation, followed by data analysis at desk through multilevel coding from the verbatim transcripts using NVivo R1 software. The data was analyzed thematically using pre-determined themes according to the MGDB theory. Emerging topics on enabling factors and constraints were captured to enhance our understanding of the complexity of cooking behavior.

**Results:**

The mothers’ mean age was about 30 years old with a comparable representation of younger and older mothers. Most mothers were housewives and accomplished secondary school level. The mothers’ intention was shown as they frequently cooked at home and allocated time for cooking. The enabling factors included their knowledge about food and nutrition, food source availability, their confidence in cooking meals and following recipes, and their motivation to keep their children healthy representing some intrinsic factors. The main constraint was the lack of skill to make snacks. The other extrinsic barriers were dependence on mobile food sellers and the availability of food kiosks that facilitated children’s snack preferences.

**Conclusion:**

The study obtained some insights that mothers had favorable cooking intention and desire, were supported with a confidence in some basic cooking skills. However, the existing constraints that encouraged the development of unfavorable children’s snacking habit were beyond the mothers’ control. A cookbook specifically for snack recipes that utilizes local ingredients may assist mother in preparing more healthier options for the children.

## Background

Children aged 2–5 years old are in a critical learning period to develop eating habits that will influence their relationships with food for a lifetime. As growing children, they tend to be very active, and independent, but also easily influenced by their surroundings such as parents and peers. Such characteristics may lead to malnutrition, including micronutrient deficiency, wasting, and overweight [1, 2]. During this age period, eating problems such as food neophobia and pickiness become major issues as they are associated with lower consumption of vegetables, fruit, and high-quality animal protein sources such as meat and fish [3, 4]. This shows the importance of the parent’s role in providing healthy meals for their children. It was a common belief that home-cooked meals were healthier [5]. However, a previous study revealed parental lack of skills in selecting more nutritious foods, their preferences for more convenient foods, and time constraints for cooking at home [6].

Cooking or culinary skill transition has been discussed, highlighting the increasing women in the workforce which causes the less time at home, the availability of affordable prepared, convenience, or ready-to-eat (RTE) foods, the knowledge and skill transferred to the next generation [7]. To understand cooking practice itself shall consider the socioeconomic inequalities, which may result in different perspective on practicality, confidence, ingredients selection, and competence in food preparation [8]. The lack of confidence to use basic cooking skills that contributes to a decline in skills has been observed in prior studies [7, 9, 10]. It was emphasized that to prepare nutritious home-cooked meals required convenient preparation, locally available food source, and reasonable cost of food [11, 12]. Purchasing pre-prepared foods from outside was seen as solution to maintain cooking at home within the available time [13]. Mothers felt that cooking from scratch was challenging and had a desire for effortless meals which grew from a general lack of motivation to cook [14].

Indonesian culinary is known for the rich taste and the complex process in cooking. Hence, this was seen as a barrier due to the knowledge on how to cook it, the limited availability of the ingredients, the limited cooking utensils, and the time required to cook [15]. Southeast Sulawesi, with the capital city of Kendari, is a province that is famous for the fish and seafood production, which shall be favorable to encourage a healthy dietary practice. A previous study in Kendari exhibited the limited knowledge among mothers on children feeding practice, that they only followed family and local community habits. This included the introduction of single dish, such as rice porridge, banana puree, or instant cereals without additional foods or ingredients as complementary foods. Although this study did not report further on cooking practice, the mothers were given nutrition education that encompassed meal preparation and homemade snacks using locally available food [16]. This shows that improving cooking skill was important to support parental feeding practice.

With the complexity of cooking and its relations with parental feeding practices being acknowledged [17], understanding the interplay of cooking behavior with its enabling factors and constraints is important especially among low-middle income mothers such as those living in Kendari. To this date, research on cooking behavior in Indonesian setting is limited. This present study provides opportunities to recognize mothers’ cooking skill, what they believe and experience about home cooking, the enabling factors, and related constraints. Additionally, this study explored cooking as the behavior of interest using Model of Goal-Directed Behavior (MGDB) in accordance with a previous study on parenting practices related to vegetable consumption [18]. This approach allows to explore the behavior in the past, thus is able to provide insights to predict the behavior and what encourages individuals to do so.

## Methods

 This study was conducted using a qualitative study design specifically utilizing focus group discussions. The study was carried out in Kendari, the capital city of Southeast Sulawesi province, Indonesia from June to July 2020. The city of Kendari had flat to hilly surfaces, as well as coastal areas. To capture varied living environments that may facilitate our understanding of the food sources in the neighborhood as well as the residents’ cooking experiences, we purposively embraced coastal areas (i.e., Abeli and Kendari sub-districts) and mainland areas (i.e., Kadia and Kendari Barat sub-districts) from all seven sub-districts in the mainland and three sub-districts in coastal areas of Kendari city. The total four sub-districts included were randomly selected.

### Participants

Eligible participants were mothers with children aged 2–5 years old who had the main role on preparing and cooking foods at home. Mothers were selected based on the cohort data of children aged 2–5 years from *Posyandu* (an integrated community health post) and screening survey conducted in each sub-district. The recruitment of the study participants was according to these criteria: (1) mothers aged 20–40 years old; (2) apparently healthy; (3) literate; and (4) children were apparently healthy and did not suffer from chronic or acute infectious diseases or congenital abnormalities. In this particular study, the children were not involved as participants. Prior to data collection, the mothers as participants were given explanation the overview of this study; those who agreed to participate signed an informed consent form.

In addition, participants fulfilled the principle of maximum variation in which varied characteristics such as education level and occupation were captured (Table 2). The initial minimum sample size adhered to a previous similar study [19]. However, the final samples included in this study was considered based on saturation of the data following the preliminary data analysis done in the field [20].

### Procedures

Data was collected through a total of six focus group discussions (FGDs). Each FGD involved 8–9 participants, lasted for approximately two hours, and took place in various venues such as the cadre’s house, *Posyandu*, or a multipurpose room at the primary healthcare center. These locations were considered reachable by the mothers and commonly used as gathering venues. The FGDs were conducted by the first author (IRN), assisted by one trained note-taker and two observers. The guide questions for the FGD were developed after a thorough literature review of the MGDB. This model was extended from the initial theory of planned behavior, with additional aspects on desire and perceived anticipated emotions that may influence desire. The MGDB concept was appropriate for exploring behavior with broader determinants such as the present study, as it allows us to explore the behavior in the past [18]. The MGDB in the present study was used to understand the experience of the mothers of young children around the multifactorial cooking behavior which consisted of cooking skill, cooking intention, cooking desire, cooking confidence, and cooking attitude.

### Discussion guide

The list of key questions used for the FGDs is presented in Table 1. All FGDs were conducted in Indonesian language and recorded using an audiotape recorder upon obtaining the participants’ consent. To enrich the data, observation through random home visits and field surveys was also carried out to provide information on home kitchen facilities, food availability at home and its surroundings, food commodities, price, vendors and markets, and other related matters on cooking.

**Table 1:** Key questions used in the FGDs

No.	Item
**Cooking skill, habits, and food/nutrition-related knowledge**	
1.	What kinds of meals and snacks do you usually prepare at home? Do you specially prepare meals and snacks for your child?
2.	Have you ever created new recipe for your child?
3.	What are the factors that you consider during cooking meals or snack for your child (taste, texture, flavor, specific ingredients, others)?
4.	When did you start getting interested in cooking and how did you first learn how to cook?
5.	What areas of your cooking skill would you like to improve?
6.	What is your water source for cooking and drinking?
7.	What do you usually do to ensure the food hygiene during cooking, preparation, and storage?
**Cooking intention**	
8.	How many times a day do you cook at home?
9.	How much time do you spend cooking each day?
**Cooking desire**	
10.	What is your main motivation to cook at home for your child?
11.	What is your desire in cooking foods for your child?
12.	How is your feeling about cooking at home?
**Cooking confidence**	
13.	How confident are you to cook meal or snack from scratch?
14.	How confident are you to cook meal or snack by following a simple recipe?
**Cooking attitude**	
15.	What do you think about cooking from scratch and cooking using store-bought ingredients?
16.	What are the factors preventing you from cooking?
17.	What do you think are the benefits of cooking?
**Other factors related to cooking (food availability and accessibility, kitchen tools and facilities, etc.)**	
18.	How is food accessibility in your household?
19.	What kinds of convenience food (i.e. instant food, frozen food, packaged food, RTE food) usually available in your home for your child?
20.	What kind of cooking equipment (cooking utensils and appliances) available in your house?

### Data analysis

Besides data analysis in the field to assist in decision on data saturation, data analysis at desk was performed through multilevel coding [20]. This was started by transcribing verbatim all the recordings from the FGDs by a trained external study team. All transcripts were checked and corrected by IRN. IRN performed the initial coding independently using NVivo R1 qualitative research software. The coding structure was discussed between IRN and JF. All other authors were involved further in the interpretation and conceptualization of the data into clearer themes and sub-themes. The data was analyzed thematically using pre-determined themes according to the MGDB theory, particularly on cooking skill, intention, desire, confidence, and attitude. The sub-themes emerged from the data after all data were coded and grouped by similar pattern. Information collected from FGDs was cross-checked with the observation and field survey results. Relevant quotations were selected to reflect the perception of the participants, which were translated from Indonesian language to English. Data are displayed in tables based on the components of cooking behavior.

## Results

A total of 33 mothers contributed in a total of six FGDs. The mothers’ mean age was about 30 years old with a comparable representation of younger and older mothers. Most mothers were housewives and accomplished secondary school level. The majority of the husbands were private employees and informal-sector workers. This concluded that the background of these households were from low-middle socio-economic. There were 22 out of 33 (66.7%) of the mothers had only one child, whereas the rest had more than one child aged 2–5 years old (Table 2). Apparently, all mothers who agreed to participate in the FGD were those who lived with nuclear family. Being in a nuclear family provided an advantage for the study to understand the main role of mothers in cooking at home.

**Table 2:** Demographic characteristics of mothers participating in the FGDs^1^

Characteristics	*n* = 33
Age (year)^2^	30.54 ± 4.85
Age group	
20–30 years	16 (48.5)
31–40 years	17 (51.5)
Education	
Secondary school	26 (78.8)
Diploma/University	7 (21.2)
Occupation	
Housewife	28 (84.8)
Merchant	3 (9.1)
Government officer	2 (6.1)
Husband’s occupation	
Private employee	13 (39.4)
Fisherman	6 (18.2)
Labor	5 (15.1)
Driver	5 (15.2)
Government officer	4 (12.1)
Number of children aged 2–5 years old in the household^2^	
1 child	22 (66.7)
> 1 children	11 (33.3)

^1^ n (%)

^2^ mean ± SD

The cooking behavior was guided by pre-determined themes such as skill, intention, desire, confidence, and attitude in cooking as shown in the following Tables 3, 4, 5, 6, and 7.

**Table 3:** Sub-themes and relevant quotations on cooking skill

Sub-theme	Quotation
Types of meals and snacks usually prepared	• *I like to cook food for my child as requested, mostly for main meals such as rice, vegetables, and fish as side dishes. However, I do not have time to make snacks for my child because we have easy access to nearby kiosks to purchase snacks like packaged drinks and biscuits.* (Mom-1 of a boy aged 31months) • *Meals for my child are like noodle or rice, with fish and veggies, while snacks such as chips ‘kerupuk’* [purchased RTE], *bread* [purchased RTE], *sometimes fried banana* [home-cooked]. *All depends on the child’s appetite.* (Mom-18 of a girl aged 40 months) • *I sometimes cook new recipes like meatball and chicken nuggets.* (Mom-4 of a girl aged 24 months)
The frequency and duration of cooking at home	• *I cook 2 times a day, sometimes 3 times a day. It depends on what to cook for the child or family*. (Mom-13 of a girl aged 57 months) • *I spend time cooking around 1–2 h, it is shorter than during holiday/weekends.* (Mom-3 of a boy aged 30 months)
Flavoring for taste	• *When I cook boiled water-spinach ‘kangkung’, I add flavoring. If I cook ‘palumara’ fish, I put a little sugar and salt for a good taste.* (Mom-30 of a boy aged 28 months) • *I usually use salt, ‘vetsin’* [flavor enhancer containing monosodium glutamate/MSG], *and a little sugar. My child looks happy and finishes the food I cooked.* (Mom-4 of a girl aged 24 months) •…*a tip of spoon or a half sachet of MSG chicken powder, only when cooking vegetables…* (Mom-32 of a boy aged 59 months) • *When I cook by myself, I can control the amount of seasoning like salt, chicken powder, sugar, MSG, herbs, and spices. So, it tastes as good as the perfect recipe for me and family.* (Mom-28 of a girl aged 59 months) • *I use coconut milk to get savory taste and a half sachet of seasoning to taste like chicken broth. My sense of taste is developed based on experience.* (Mom-22 of a boy aged 59 months) • *When I cook ‘palumara’ fish, I use lots of onions, garlic, and shallot, add brown sugar as part of seasoning to make it more delicious.* (Mom-9 of a girl aged 59)
Cooking skill for making snacks to be improved	• *Because my children like snacking very much, making snacks at home, especially the healthy snacks for them needs to be improved. Sometimes they like to ask for cakes because in front of our house there is this ‘warung’* [kiosk]. *My kids like cookies and cakes.* (Mom-14 of a boy aged 50 months)
New recipe development	• *I wish I could make a new recipe, but no ma’am, I cook original recipe that came from my own mind. My child doesn’t like new food. Sometimes I only cook the leftover fish and modify it from boiled to fried with additional flavor like ketchup and so on.* (Mom-28 of a girl aged 59 months) • *My new recipe is ‘abon telur’* [shredded eggs]. *It is made of fried chicken eggs and mixed with sweet potatoes. The children really like to eat this, because the taste is savory and crunchy from the sweet potatoes.* (Mom-25 of a boy aged 59 months)
Food/nutrition-related awareness	• *To get more balanced nutrient intake for the children, food hygiene needs to be paid attention to. Mothers should know how to wash foods from markets and fish auctions thoroughly so the germs would be removed, so the children can be healthy.* (Mom-22 of a boy aged 59 months) • *… of course, that is why I cook myself. If we buy food from outside, we don’t know the food hygiene, whether the seller washes it first before cooking, or other ingredients whether it is safe or not, whether the handlers are clean or not.* (Mom-11 of a boy aged 24 months) • *I usually buy some gallon refill drinking water for cooking and drinking. The price is IDR 6,000 per gallon. I boil it first before drinking.* (Mom-22 of a boy aged 59 months)

**Table 4:** Sub-themes and relevant quotations on cooking intention

Sub-theme	Quotation
The availability of cooking tools and kitchen facilities and how to utilize them	• *If the ingredients and cooking tools are available and I know the recipe, these can help me cook easily*. (Mom-25 of a boy aged 59 months) • *I still borrow my parents’ equipment except the stove.* (Mom-21 of a girl aged 33 months) • *I have my own kitchen equipment*. (Mom-10 of a boy aged 30 months) • *Spoon, plate, pan, stove, not everything is mine, some are my parents’ properties. If suddenly the ‘LPG’ [gas] runs out, I don’t know how to put it on my gas stove, I am afraid it will explode.* (Mom-8 of a boy aged 50 months)
Food sources surrounding the house	• *It is easy to get moringa, papaya from back/front yard, and I grow hydroponics vegetables too, I grow mustard greens, kale, I put them on the side of the house.* (Mom-9 of a girl aged 59 months) • *I can take spinach, kale, bananas, papayas freely*. (Mom-17 of a boy aged 24 months) • *From my front yard, I can take ‘katuk’ leaves, ‘kedondong’(tawoloho) leaves, ‘licin’ leaves.* (Mom-16 of a girl aged 38 months) • *Wild veggies like ‘tekoka’ from the forest, and also ‘leunca’.* (Mom-7 of a boy aged 53 months) • *It’s easy to go everywhere, my house is close to the fish auction place, I usually buy it for 3 days, I keep it in my refrigerator.* (Mom-31 of a girl aged 24) • *I am just waiting for ‘Ina/Ode Penjual Ikan’and ‘Mas Sayur’* [mobile vegetables seller]. (Mom-30 of a boy aged 28 months) • *So many kiosks here, we can find soy sauce, tomato sauce, a dish like ‘Nasi Kuning’* [yellow rice with some side dishes] *and cooked vegetables that are ready to eat*. (Mom-27 of a girl aged 38 months) • *There are ‘Warung Bakso’* [meatball soup kiosks], *also those selling cooked vegetables, noodle soups, located on the side of the road; it is easily accessed.* (Mom-28 of a girl aged 59 months)
Barriers to cooking at home	• *I can’t cook properly. When I don’t have enough money for buying food ingredients and I don’t find myself some free time in the kitchen, I will let my child buy meatball soup from the kiosk.* (Mom-1 of a boy aged 31 months) • *Because my house is up in the mountains, it would be difficult to cook if ‘Mas Sayur’and ‘Ina/Ode Ikan’don’t operate. So, on that day I don’t cook.* (Mom-5 of a girl aged 33 months)

**Table 5:** Sub-themes and relevant quotations on cooking desire

Sub-theme	Direct quotations
Motivation to cook	• *I like to cook, I am happy when cooking, I always cook for my child and family*. (Mom-6 of a girl aged 33 months) • *When I don’t cook, I see my family in trouble. Cooking is my hobby. I cook every day and like to try new recipes although it fails most of the time*. (Mom-4 of a girl aged 24 months) • *My child likes to eat snacks. He had a great appetite. I cook more often than buy takeout to get healthier food. I really care about my child than my husband because my husband is rarely at home as he is a fisherman.* (Mom-8 of a boy aged 50 months) • *I desire to cook food like the street vendor sells, like ‘terang bulan’* [sweet cakes] *that is cheap and yummy, but when I tried to cook it, it failed, and the cake was burnt. I want to see my child happy with the food that I make it with the taste that is similar to the street food vendor*. (Mom-9 of a girl aged 59 months)
Interest and family influence	• *Eating meals together with family has many other benefits, like trying to offer small amount of veggies that other family members can help.* (Mom-25 of a boy aged 59 months) • *After getting married, my first interest was cooking for my husband*. (Mom-4 of a girl aged 24 months) • *I started to have an interest in cooking like my mom, after I got married. She taught me some recipes that I cook until now. My sister taught me too.* (Mom-24 of a girl aged 59 months) • *My first real enjoyment of cooking came when I was in junior school. It came from only cooking rice and instant noodles with some boiling water. My mom taught me.* (Mom-8 of a boy aged 50 months) • *I finally can cook like my mother. She taught me to cook. She’s a very good cook. When I have the right ingredients, I feel that now the food I cook tastes slightly the same as my mom’s.* (Mom-27 of a girl aged 38 months)

**Table 6:** Sub-themes and relevant quotations on cooking confidence

Sub-theme	Quotation
Cooking with instructions from the recipes	• *If the ingredients are available, I have the confidence to place the ingredients based on the recipe steps. I have received food orders (meals and/or snacks) from my church charity activity, and I can handle it. I do the cooking myself.* (Mom-9 of a girl aged 59 months) • *I usually try it first, but I’m sure I can follow the new recipe and follow the instructions*. (Mom-7 of a boy aged 53 months)
Cooking from scratch or the use of fresh ingredients	• *I cook every day, I buy veggies, fish, and herbs from a traditional market or ‘Mas Sayur’.* (Mom-18 of a girl aged 40 months) • *I love to cook from fresh ingredients. I usually replace sweet soy sauce with brown sugar.* (Mom-19 of a girl aged 48 months)
Opinions on the taste of food cooked by the mother	• *My child said it was delicious.* (Mom-5 of a girl aged 33 months) • *I’m pretty sure that my food is always tasted by my children. I cook using chicken powder and the food is yummy and very tasty.* (Mom-15 of a girl aged 44 months) • *Other people’s cooking does not necessarily have the same taste as mine.* (Mom-28 of a girl aged 59 months) • *Because my child likes my soup, I’m sure I can say, and I guarantee that he can’t find the soup as delicious as my cooking.* (Mom-8 of a boy aged 50 months)

**Table 7:** Sub-themes and relevant quotations on cooking attitude

Sub-theme	Quotation
Perception towards instant food/ processed food and child’s health	• *I don’t cook instant noodles because my child has an allergic reaction to it.* (Mom-21 of a girl aged 33 months) • *Some processed foods can cause illnesses, but we can’t avoid them from our daily menu. So, I tend to mix or use both convenient products and fresh ingredients.* (Mom-25 of a boy aged 59 months) • *My child is allergic to eggs, her skin would have rash and become itchy*. (Mom-24 of a girl 59 months) • *My child can’t eat tuna, only for tuna, it can cause rash and swelling all over her skin*. (Mom-9 of a girl aged 59 months) • *… no drinking soda drinks and artificially sweetened soft drinks, or other sweetened packaged drinks. He will be sick, catch flu and cough.* (Mom-14 of a boy aged 50 months)
Perceived benefits of cooking at home	• *Cooking at home is more efficient, and cheaper than eating out.* (Mom-4 of a girl aged 24 months) • *Healthier because it is from fresh ingredients. I can cook in small quantities and can set the cooking time.* (Mom-1 of a boy aged 31 months) • *My family can get more nutritious food if I prepare it myself*. (Mom-8 of a boy aged 50 months) • *Food hygiene is guaranteed when you cook by yourself.* (Mom-6 of a girl aged 33 months)
Strategies for the leftover foods	• *I will ask my child to eat it again after playing* (Mom-25 of a boy aged 59 months) • *I will eat the leftover, or I will persuade him to eat it again later and put the plate in the dining table with a cover (*Mom-6 of a girl aged 33 months*)*

### Cooking skill

Usually, mothers cooked main meals 2–3 times a day for all family members. As for snacks, the mothers preferred to buy from the kiosk instead of cooking by themselves because it was considered more efficient. The average amount of time spent cooking food was 1–2 hours and could take longer at weekends. Most mothers preferred to cook food from fresh ingredients, yet some mothers also used instant foods. They mostly used salt, sugar, store-bought sauces (e.g., soy sauce, ketchup), and flavor enhancers in their cooking, including the food for their children in limited amounts. They mentioned that they needed to improve their skill in cooking snacks because their children liked to eat snacks more than main meals. The preference of their children also encouraged the mothers to come up with new menus or recipes, such as *abon telur* (shredded eggs), nuggets, and meatballs. When cooking, the mothers considered food hygiene, including the usage of boiled water since the water sources may come from mountain springs, gallon refills, or tap water. They also perceived essential foods for children which included rice, fish, *tempe*, and vegetables. The knowledge of food and nutrition for young children was generally obtained from midwives or *Posyandu* cadres.

### Cooking intention

Most mothers had the intention to cook for their young children but were restricted by the limited knowledge and skills on how to cook some particular foods. The availability of cooking tools and kitchen facilities also determined the level of practicality of cooking at home. Although they had the willingness to cook, the ingredients sometimes were not available despite some greens being easily accessible. Some mothers lived in a neighborhood that was near a forest where wild edible plants could be found. Some had gardens in their homes or in the neighborhood that provided vegetables such as moringa leaves, papaya leaves, bananas, *tekoka*, *leunca*, sweet potato, mushrooms, *tawaloho*, spinach, and mustard greens. Some also lived near a store or fish auction place. On top of that, time constraint was frequently mentioned by the mothers. Limited financial resources to purchase ingredients, limited access to fresh food markets, and modest kitchen facilities were mentioned particularly by mothers with low economic status or mothers who lived in mountainous areas. Some mothers in more urbanized areas mentioned the easy access to kiosks or stores that sold cooked foods and RTE snacks.

### Cooking desire

The majority of the mothers showed their desire in cooking since they believed that home cooking was essential to make all family members happy by getting their tummy feeling full. Naturally, cooking practice had become a part of their daily life. Most mothers gained exposure to cooking from their mother (the child’s grandmother). Many of them stated that cooking had become their habit after they got married; some also had been exposed to cooking since their school age. Cooking for their children was seen as a priority. The mothers portrayed a good feeling when they watched their children having a great appetite when eating home-cooked foods. Nonetheless, they were concerned because they could only cook main meals and had limited options for home-cooked snacks. The mothers often mentioned their desire to be able to create healthy snacks for their children. Some mothers also described cooking as their hobby, and they had fun doing experiments and renewing their own recipes.

### Cooking confidence

The mothers were mostly confident in cooking, as they believed their own cooking results were delicious and had different, unique tastes that fit their family members’ preferences. Taste was perceived as the greatest strength. If they were given recipes, they were also confident that they would be able to cook by following the instructions if the ingredients were available and well-prepared. Some mothers also ran a catering business and received orders to provide food for events in the village. Thus, cooking from scratch using fresh ingredients was considered not a problem for some mothers.

### Cooking attitude

To reduce the risks of getting a disease among their children, most mothers attempted to cook using fresh ingredients rather than instant foods. Some mothers were concerned about instant noodles consumption and other foods such as eggs which might trigger an allergic reaction. This pushed the mothers to be more creative in cooking. Moreover, mothers believed in the benefits of cooking at home for the children’s health and for the family’s economy as the cost would be cheaper than eating out. In addition, most mothers disapproved of complaints about their cooking, as well as leftovers. Nonetheless, they preferred having leftovers to getting complaints about their food, since leftovers could still be consumed later by the child or the mothers themselves.

### Food sources around the neighborhood and other constraints

Some enablers related to cooking behavior are presented above. However, Tables 3, 4, and 7 also highlight some constraints that are either intrinsic or extrinsic factors. In terms of cooking skills, some mothers faced challenges in making snacks due to their limited ability. This was modified by an external factor i.e., the presence of kiosks as the significant food source for child snacks around the neighborhood. A sense of high reliance on the use of flavoring enhancers in their cooking may be regarded as a manifestation of presenting tasty food for the family as this characteristic was highly valued in the foods provided at home. In addition, picky eating children complicated the mothers’ already limited cooking skills (Table 3). This complexity was shaped further by some unfavorable cooking attitudes because some of their children also had allergic reactions to foods. Thus, mothers must be consistently aware of cooking some foods safe from any allergic-causing ingredients (Table 7), suggesting that mothers’ knowledge on this matter must be kept updated. The cooking intention of these mothers was also constrained by the availability of modest cooking tools and kitchen facilities that potentially hindered experiments with new or modified recipes. In addition, dependence on mobile grocery sellers limited mothers’ creativity in cooking despite many other food sources being available in their surroundings. This may represent mothers’ lack of “recipe vocabulary” (Table 4).

The field survey took place at three traditional markets and three food shops that were selected randomly. It was evident that snacks were displayed in front of the shops, which meant that children can easily spot them. This was aligned with the concern raised by the mothers as their children preferred snacking. Meanwhile in the traditional markets, fish and vegetables were abundant and accessible. This showed the availability of locally nutrient dense food source. Another observation through home visits also confirmed the availability of simple and basic cooking tools and utensils, such as frying pan or wok, pot, steamer, and gas stove. This highlighted the limited variation of cooking techniques among the mothers.

## Discussion

This qualitative study provides some insights into the cooking behavior among mothers with young children, particularly who lived in low-middle socio-economic circumstances. To the best of our knowledge, this is the first study to explore cooking behavior using MGDB model inspired from the study of Baranowski et al. [18] on parenting practices related to vegetable consumption. Using this model, we conceptualize cooking skills to be shaped by cooking intention in which it relates to cooking desire. Cooking desire is a manifestation of the interplay of cooking confidence, the attitude towards cooking, and all enablers and constraints faced by the mothers both intrinsically and extrinsically. Overall, this model allows us to understand that the cooking desire of the mothers was strongly related to the willingness of the mothers to provide healthy meals for their children.

The major findings in this present study included the decent knowledge among mothers that was obtained from the health workers and their elder relatives. The mothers also had good intentions in cooking, although some of them had financial constraints. The concept of cooking skills encompassed the aspect of knowledge of food and nutrition, as well as the ability to conceptualize the end product and how to process it (from raw ingredients or convenient products) [21]. We found that mothers had barriers especially when making snacks. They acknowledged the type of snacks consumed frequently by the children and wanted to create similar food at home with healthier ingredients. Despite being able to conceptualize the snack they wanted to make, they had limited skill and ability to recreate it, as the skill they had was mainly to cook main meals.

Snacking among children was one of the major concerns among the mothers since the types of snacks consumed were not nutrient-dense. Nonetheless, Indonesian dietary guidelines stressed the importance of healthy snacking, which could be introduced during complementary feeding. The recommendation specifically for children aged 2–5 years old is to have three times a day of main meals, and snacks two times a day between the main meals. This recommendation also endorsed family meals and the limitation of processed foods that are high in sugar, salt, and fat [22]. Our finding on the willingness of the mothers to provide homemade meals and snacks believed to be healthier and safer was in line with the existing national dietary recommendations.

The mothers demonstrated a good intention as shown in the frequency of cooking in a day and time spent for food preparation, which was supported by a previous study that the time spent in the kitchen may be up to one hour and the time on weekends were more flexible [5]. Most of the mothers were housewives without jobs, which may encourage them to spend more time in cooking as there were fewer barriers related to working away from home. Furthermore, a study in France found that the COVID-19 pandemic allowed parents to allocate more time to preparing food and they were willing to maintain this practice [23]. For the mothers who came from low-middle economic backgrounds, cooking at home was seen as economically beneficial. Purchasing food away from home was perceived as more expensive, as supported by a review that found budget control as one of the motivators for home cooking [24]. Buying takeout foods was more common among families with higher incomes [15]. It was common in urban areas for the mothers to only cook rice at home, while the side dishes such as vegetables and protein source foods were purchased from home-style food kiosks (i.e., *warteg*) for such an affordable price [25, 26]. Nevertheless, this practice did not seem to be familiar in our study area.

Another finding in this study is that cooking desire among these mothers was aimed to maintain their child’s health. This was also related to the cooking attitude, in which the mothers preferred cooking from fresh ingredients, although some still used convenience food such as instant packaged food or chicken powder for flavor enhancer. It was a common belief that homemade meals were healthier [5] as it was more likely to contain fruits and vegetables, thus may contribute to better diet [27]. The environment of these mothers supported them to prepare healthy food since their houses were close to a forest or garden that provided vegetables. Having vegetables and fruits accessible at home was believed to encourage parents to include them in the meal menu [28]. However, a remaining issue for the mothers was the child’s tendency of picky eating. Only a few who were courageous to try creating new menu for their child, which may require confidence in cooking. Individuals with high confidence in food skill were likely to have more control over family food behavior. Whereas low confidence in food skill was associated with more impulsive purchasing of unhealthy food [9]. Despite the narrative of the mother’s confidence in following new recipes, this study did not particularly measure the degree of food skill confidence.

Snacking over meals was frequently mentioned and raised as a concern. Earlier it was stated that the mothers had a supportive environment. As food availability and accessibility were frequently discussed, it actually could be both facilitators and barriers to home cooking [24]. In the context of snacking, we found that mothers preferred purchasing RTE snacks available in the small shops around the house. A qualitative study in Jakarta portrayed how a neighborhood was surrounded with various food shops [25], which may be experienced in the present study area as well. This shows that easy access to food shops could be a barrier to home cooking. A previous study highlights how Indonesian foods are complex and time consuming to make [15]. While mothers from higher socioeconomic status can hire domestic helps to assist them preparing food, have more equipment like baking tools [15], and can utilize the advanced technology such as microwave [10], the mothers in our study setting were not able to do so.

Prior studies provide promising evidence that cooking intention predicts cooking skill [29]. Furthermore, we can manipulate intention as it can be modeled through training that facilitates experiential learning to activate the skill development [30]. The present study was designed to understand the cooking behavior of mothers of young children prior to the development of a cookbook. Understanding the complexity of the cooking behavior will assist the cookbook development process to be relevant with the enabling factors and sensitively addressing the constraints faced by the mothers. The present study suggests the need to develop a cookbook specifically for snack recipes that utilizes local ingredients from the surroundings and incorporates simple cooking techniques with modest cooking tools. The use of the cookbook needs to be further studied to design an efficient and effective cooking educational program. To this day, there was not much information on nutrition educational program targeting cooking behavior. Only in 2022 the Ministry of Health Republic of Indonesia launched a cookbook with the main target of pregnant women and under-five children. Nonetheless, the recipes in this book represented all regions of Indonesia, incorporating local ingredients from respective regions. The present study found the need to facilitate home cooking specifically on healthy snack recipes that are acceptable for the children.

The strengths and limitations of this study are as follows. This is the first study in Indonesia to explore cooking behavior using the MGDB model, capturing skill, intention, desire, confidence, and attitude in cooking among mothers of young children. The MGDB theory itself emphasized the importance of desire, which previous studies on cooking did not highlight. In this study, triangulation of data sources was not able to be performed due to limited study permission during the COVID-19 pandemic. Views from other family members were not able to be obtained to enrich the experience of cooking at home as mentioned by the mothers.

## Conclusion

Using MGDB theory, the present study captured the cooking behavior that involved skill, intention, desire, confidence, and attitude in cooking. Interest and family influence on cooking, willingness to cook, desire to keep their children healthy, belief in homemade food to be a healthier option, and local food availability were some of the enabling factors. On the other hand, mothers faced limitations in their skills to make snacks at home. With the vast availability and accessibility of food shops, packaged food and drinks were preferred as snacks for the children despite the mothers being deeply concerned. The interplay of these factors made cooking behavior a complex matter, especially with the presence of enabling factors and constraints when it comes to the child’s snacking habit. With the existing cookbook and menu plan recently launched in 2022 by the Ministry of Health Republic of Indonesia, the present study may stimulate further efforts to increase the relevance of the cookbook, particularly on simple snack recipes that utilize local ingredients specifically designed for mothers with 2–5-year-old children in Kendari city.

## Data Availability

The datasets generated and/or analysed during the current study are not publicly available due to privacy and ethical concerns, neither the data nor the source of the data can be made available, but are available from the corresponding author on reasonable request.

## Abbreviations

FGDs: focus group discussions
MGDB: Model of Goal-Directed Behavior
RTE: ready-to-eat
